# Chronic neuropathic facial pain after intense pulsed light hair removal. Clinical features and pharmacological management

**DOI:** 10.4317/jced.52520

**Published:** 2015-10-01

**Authors:** Cosme Gay-Escoda, Gabriela Párraga-Manzol, Alba Sánchez-Torres, Gerardo Moreno-Arias

**Affiliations:** 1 MD, DDS, MS, PhD, EBOS. Chairman and Professor of the Oral and Maxillofacial Surgery Department, School of Dentistry, University of Barcelona. Director of Master’s Degree Program in Oral Surgery and Implantology (EHFRE International University/FUCSO). Coordinator/Researcher of the IDIBELL Institute. Head of Oral and Maxillofacial Surgery and Implantology Department of the Teknon Medical Center, Barcelona (Spain); 2 DDS. Fellow of the Master of Oral Surgery and Orofacial Implantology. School of Dentistry, University of Barcelona (Spain); 3 DDS. Fellow of the Master of Oral Surgery and Orofacial Implantology. School of Dentistry, University of Barcelona (Spain); 4 MD, PhD. Dermatology Department, Centro Médico Teknon (Barcelona), and Espitau Val d’Aran (Vielha, Lleida), Spain

## Abstract

Intense Pulsed Light (IPL) photodepilation is usually performed as a hair removal method. The treatment is recommended to be indicated by a physician, depending on each patient and on its characteristics. However, the use of laser devices by medical laypersons is frequent and it can suppose a risk of damage for the patients. Most side effects associated to IPL photodepilation are transient, minimal and disappear without sequelae. However, permanent side effects can occur. Some of the complications are laser related but many of them are caused by an operator error or mismanagement. In this work, we report a clinical case of a patient that developed a chronic neuropathic facial pain following IPL hair removal for unwanted hair in the upper lip. The specific diagnosis was painful post-traumatic trigeminal neuropathy, reference 13.1.2.3 according to the International Headache Society (IHS).

** Key words:**Neuropathic facial pain, photodepilation, intense pulse light.

## Introduction

Nowadays laser and light sources are used worldwide for different treatments especially for permanent or prolonged hair removal. Their easy and fast application, non-invasiveness, and few minimal and transient known side effects have made them very popular (1).

Although Intense Pulsed Light (IPL) photodepilation is an effective and safe hair removal method, caution should be considered by practitioners and patients to avoid permanent side effects (2,3).

Chronic facial pain after a hair removal treatment with IPL has not been reported. The authors present a case of chronic neuropathic facial pain after IPL hair removal.

## Case Report

**Figure 1:**
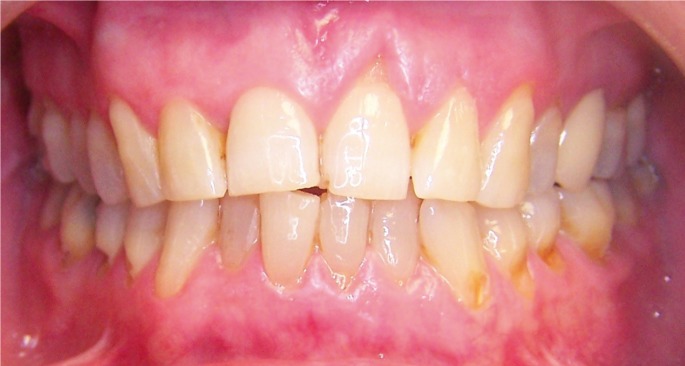
Clinical examination: intraoral frontal view.

**Figure 2:**
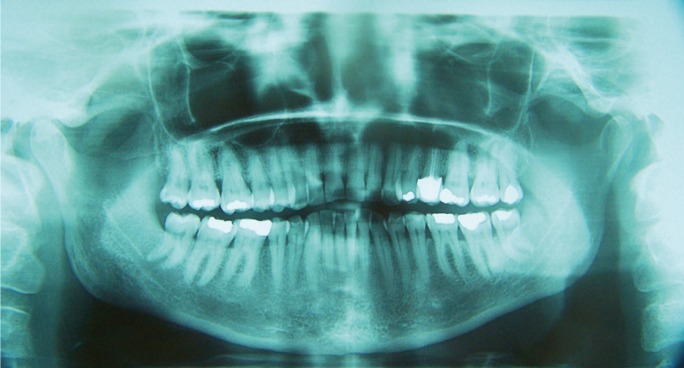
Panoramic dental radiology.

A 58-year-old Caucasian female was seen at the Orofacial Pain Unit of the Oral and Maxillofacial Master Degree Program at the University of Barcelona with a 5 year history of orofacial pain following IPL hair removal for unwanted hair in the upper lip. Two different wave lengths and IPL sources were used during the procedure (650 nm cut-filter, Lovely, Alma, Israel and 695 nm cut-filter, Quantum, Israel). The patient referred that from the fourth IPL treatment session, she started suffering severe (8/10 on a Visual Analog Scale (VAS)), constant pain and numbness localized at the anterior hard palate, the upper central incisors, the buccal side mucosa and sub-nasal area. She reported that non-opioid analgesics such as ibuprofen were unsuccessful in controlling the pain. During the initial visit at our unit, the patient signed an informed consent to be attended there, underwent a thorough anamnesis and clinical examination that included panoramic X-rays (Figs. 1,2) and periapical radiographs of the painful area. These examinations did not show any relevant abnormalities. Her personal medical history revealed no toxic habits, acetylsalicylic acid intolerance, chronic rhinitis (2004), hysterectomy (2005), kidney lithiasis (2008), osteoarthritis and primary essential hypertension (controlled with diet). Current medication included calcium 60 mg QD and paracetamol (acetaminophen) 1 gr on demand. The whole patient interview was performed by a dentist with advanced training in the management of Orofacial Pain conditions following the guidelines of the American Academy of Orofacial Pain and the International Headache Society (IHS) (4). Although the symptoms were bilateral, all other characteristics agreed with diagnostic criteria of a V2 painful post-traumatic trigeminal neuropathy (IHS 13.1.2.3). Nasopalatine nerve provides innervation to palatal gingiva and mucosa, and the nasal septum. This is a unique case because nasopalatine nerve is located at the midline and the affected structures are bilateral. Initial treatment began with educating the patient to the condition and teaching her some basic physical self-regulating strategies to reduce sympathetic nervous system up-regulation (diaphragmatic breathing). A series of blood tests (complete blood count, kidney and liver function, full ionogram, lipid panel and fasting glucose levels) were requested in order to obtain baseline parameters before the administration of any medication. All results were normal.

Medical management began with amitriptyline 25 mg QHS with a discrete improvement of pain intensity after 4 weeks of treatment. This medication was then associated with clonazepam (Rivotryl, Roche Farma, Madrid, Spain) 0.25 mg BID. Two months later, the patient referred substantial improvement (1-2/10 VAS) of pain in the nasal area and upper anterior teeth, complaining only of a mild discomfort in the anterior hard palate. Clonazepam was adjusted to 0.25 mg TID. Three months later the patient reported gastric distress and therefore amitriptyline was changed for duloxetine 30 mg QD and omeprazole 20 mg QD was added to the pharmacologic protocol. This change in the medication did not provoke any substantial alteration in pain control; however, gastric distress was controlled satisfactory. This medication protocol has been maintained over a two year follow-up period in which the patient has remained virtually asymptomatic, reporting no major side effects from the medication use.

## Discussion

Definitive diagnosis of chronic orofacial pain is challenging. However, this situation is relatively frequent for dentists because persistent and chronic pain is more common in the head and neck region than in any other part of the body. Following identical injuries, the onset of neuropathic pain and its characteristics varies individually. Such variability is probably due to a combination of environmental psychosocial and genetic factors. Some patients develop chronic pain following a negligible nerve trauma such as root canal therapy. Besides, it is widely known that third molar extractions may lead to disturbed sensation in the lingual or inferior alveolar nerve for varying periods. Some other procedures as dental implant placement or orthognathic jaw surgery may produce permanent sensory dysfunction. Nevertheless, the incidence of chronic pain is still unclear (5).

It is unlikely that our patient already had an undiagnosed chronic facial pain and that it was overlooked before IPL hair removal treatment since dental therapy (endodontic treatment) in the painful area was performed several years before IPL use, with no discomfort and/or pain reported then by the patient. Although IPL photodepilation is an effective and safe hair removal method, caution should be considered to avoid permanent side effects (2,3). IPL photodepilation procedure is painful. The majority of patients experience mild pain during treatment administration but it should always be tolerable especially if sensitive areas such as the upper lip are being depilated. Administration of local anaesthesia is not usually recommended for adult patients because pain is the best early warning system to prevent side effects caused by heat destruction. If the patient complains about intolerable pain, the risk of adverse effects is high (3,6).

Despite the specificity of light sources used today, complications may still appear. Although some of the complications are laser related, many are still caused by an operator error, either in consciousness or by a postoperative mismanagement (3). The use of light and laser devices by medical laypersons poses a real threat to public health and the potential damage should not be underestimated (7). Fortunately, most side effects associated to IPL photodepilation are transient, minimal and they disappear without sequelae. Foliculitis, pigmentary alterations relating to skin color, blisters and crusts, erythema and edema are the most common adverse effects (6). Concretely, Fodor *et al.* (2) mentioned that hypopigmentation is less frequently reported in the literature com-pared with hyperpigmentation. Likewise, Hammes *et al.* (7) performed a retrospective study to evaluate treatment errors arising from laser and IPL treatments by laypersons. A standardized survey and a photo-documentation were given to the participants. From a total of 50 patients, the reported complications were pigmentation changes (81.4 %), scarring (25.6 %), textural changes (14 %) and inadequate information without physical injury (4.6%). Another known side effect is called “paradoxical” effect (more hair in close proximity to the treated area) (1,2,6,7). The mechanism for paradoxical hypertrichosis is not known. Moreno-Arias *et al.* (6) believes that sublethal doses of IPL may have induced activation of dormant follicles.

Even though permanent side effects exceptionally occur, they are possible and seem to be related to use of high fluences (superficial burning, isolated vesicles), infection (scar formation), and dark skin (pigmentary alterations, especially in phototypes higher than IV) (6). This is in accordance with the results found in the study performed by Hammes *et al.* (7) that reported treatment errors done in 50 patients that consisted in excessive energy application (62.8 % of patients), wrong device used for the indication (39.5 %), a deep tan or a skin tone too dark for the intervention (20.9 %), inadequate cooling (7%) and inadequate information (4.6%). Only a few permanent side effects have been reported in the literature. Fodor *et al.* (2) reported one case of hypopigmentation and one case of leukotrichia. Moreover, Moreno-Arias *et al.* (6) obtained a minimal atrophic scar in the submandibular area. They also mentioned that one patient experienced persistent local heat sensation (not burning) without erythema or other symptoms. Sperber *et al.* (8) reported a 21-year-old woman who experienced a severe blistering eruption meanwhile Shin *et al.* (9) exposed a case of a 41-year-old woman who developed vitiligo on the cheek and the mandibular area following an IPL treatment.

Proper patient selection is mandatory for reasonable success rates. It is up to the practitioner to determine whether each individual patient is suitable for laser treatment and whether the correct technology and skills are available for treatment (3). Physicians should always make the indication for the treatment and are responsible for setting the machine for each individual patient and treatment. The type of laser or IPL and their specific parameters must be adapted to the indication (such as the vessels characteristics or the Fitzpatrick skin type).

Treatments should start on a test patch and a treatment grid could improve accuracy. It is important to carefully observe the treated test spot for at least 5 minutes before proceeding with the full treatment and before each session. The treatment parameters should be adjusted according to the skin response from the previous session. Cooling has become an integral part of laser treatments (cryogen spray, cold sapphire contact handpieces or air pre-cooled and blown across the skin surface). These devices promote rapid epidermal cooling to lower temperatures without affecting the target (3).

## Conclusions

The authors did not found scientific evidence regarding the relation between a facial neuropathic pain and IPL treatment. In order to prevent accidents and unnecessary risks, a proper training of specialist staff regarding security and protection is required. This is mandatory for the specialist that applies treatments with different light sources to know the necessary prevention measures to avoid complications.

Detailed explanations about the possible side effects, proper selection of patients, cooling the treated area, and avoiding sun ex-posure between treatments may also contribute to a higher patient satisfaction rate and fewer complications.
